# Diversity of nonribosomal peptide synthetase and polyketide synthase gene clusters among taxonomically close *Streptomyces* strains

**DOI:** 10.1038/s41598-018-24921-y

**Published:** 2018-05-02

**Authors:** Hisayuki Komaki, Kenta Sakurai, Akira Hosoyama, Akane Kimura, Yasuhiro Igarashi, Tomohiko Tamura

**Affiliations:** 1 Biological Resource Center, National Institute of Technology and Evaluation (NBRC), Chiba, 292-0818 Japan; 2 NBRC, Tokyo, 151-0066 Japan; 3 Biotechnology Research Center and Department of Biotechnology, Toyama Prefectural University, Toyama, 939-0398 Japan

## Abstract

To identify the species of butyrolactol-producing *Streptomyces* strain TP-A0882, whole genome-sequencing of three type strains in a close taxonomic relationship was performed. *In silico* DNA-DNA hybridization using the genome sequences suggested that *Streptomyces* sp. TP-A0882 is classified as *Streptomyces diastaticus* subsp. *ardesiacus*. Strain TP-A0882, *S. diastaticus* subsp. *ardesiacus* NBRC 15402^T^, *Streptomyces coelicoflavus* NBRC 15399^T^, and *Streptomyces rubrogriseus* NBRC 15455^T^ harbor at least 14, 14, 10, and 12 biosynthetic gene clusters (BGCs), respectively, coding for nonribosomal peptide synthetases (NRPSs) and polyketide synthases (PKSs). All 14 gene clusters were shared by *S. diastaticus* subsp. *ardesiacus* strains TP-A0882 and NBRC 15402^T^, while only four gene clusters were shared by the three distinct species. Although BGCs for bacteriocin, ectoine, indole, melanine, siderophores such as deferrioxamine, terpenes such as albaflavenone, hopene, carotenoid and geosmin are shared by the three species, many BGCs for secondary metabolites such as butyrolactone, lantipeptides, oligosaccharide, some terpenes are species-specific. These results indicate the possibility that strains belonging to the same species possess the same set of secondary metabolite-biosynthetic pathways, whereas strains belonging to distinct species have species-specific pathways, in addition to some common pathways, even if the strains are taxonomically close.

## Introduction

A large number of bioactive secondary metabolites have been found from actinomycetes^1,2^. In past years, each secondary metabolite producer was taxonomically identified at the species level based on morphological, cultural, physiological and chemical features. Consequently, correlation data between each species and its secondary metabolites are steadily being accumulated. For example, *Streptomyces griseus*, *Streptomyces avermitilis* and *Streptomyces tsukubensis* are well known to produce streptomycin, avermectin and tacrolimus, respectively^3–5^. However, taxonomic position of producing strains of new secondary metabolites are usually determined at the genus level based on their 16S rRNA gene sequences, while species-level assignment is not always done in the field of natural product research. Although species-level classification of secondary metabolite producers gives crucial information for researchers who are seeking new microbial compounds, relationship between species names and secondary metabolites is unclear for most cases.

Genome analyses of actinomycetes are revealing that various biosynthetic gene clusters (BGCs) for secondary metabolites are encoded in their genomes and about half to three quarters of the clusters are associated with nonribosomal peptide synthetase (NRPS) and polyketide synthase (PKS) pathways^6^, which suggests that nonribosomal peptides, polyketides and their hybrid compounds are the major secondary metabolites of actinomycetes. These compounds often show pharmaceutically useful bioactivities, and many have been developed into various drugs such as antibiotics, anticancer agents, and immunosuppressants. Hence, recently, genome analysis focused on NRPS and PKS gene clusters is often employed to evaluate actinomycete strains for their ability of secondary metabolite production^7–10^.

A marine-derived *Streptomyces* sp. TP-A0882 produces butyrolactol^11^. We recently identified the gene clusters responsible for butyrolactol and thiazostatin biosynthesis in this strain using whole genome analysis^12^. In the present study, we sequenced the genomes of three type strains taxonomically closely related to strain TP-A0882, and conducted *in silico* DNA-DNA hybridization (DDH) to identify this strain at the species level. We further analyzed secondary metabolite-BGCs (smBGCs) such as NRPS and PKS gene clusters in each of the genomes to elucidate the diversity of secondary metabolite-biosynthetic pathways among the taxonomically close species and provide information useful for researchers screening *Streptomyces* strains for new compounds.

## Results

### Taxonomic identification of butyrolactol-producing *Streptomyces* sp. TP-A0882

The 16S rRNA sequence of *Streptomyces* sp. TP-A0882 showed >99% nucleotide similarity to those of *S. diastaticus* subsp. *ardesiacus* NRRL B-1773^T^ (99.9%, 1464/1465), *S. coelicoflavus* NBRC 15399^T^ (99.4%, 1455/1464), and *S. rubrogriseus* LMG 20318^T^ (99.0%, 1448/1462). Next, we sequenced the genomes of *S. diastaticus* subsp. *ardesiacus* NBRC 15402^T^, *S. coelicoflavus* NBRC 15399^T^, and *S. rubrogriseus* NBRC 15455^T^ and compared them with the previously sequenced genome of *Streptomyces* sp. TP-A0882 to estimate their DNA-DNA relatedness values. As shown in Table 1, the DDH estimate for the comparison between *Streptomyces* sp. TP-A0882 and the *S. diastaticus* subsp. *ardesiacus* type strain was 94.4%. Because the probability that the DDH estimate value exceeds 70% was calculated as 97.1% (Table 1), these two strains were confirmed to belong to the same species. On the other hand, the DDH estimates between *Streptomyces* sp. TP-A0882 and the other taxonomically close species were lower than 46%. Therefore, we identified *Streptomyces* sp. TP-A0882 as *S. diastaticus* subsp. *ardesiacus*.

**Table 1:** Genome sequencing and digital DNA-DNA hybridization (DDH) values estimated by GGDC 2.1.

Strain	Reads (Mb)	No. of scaffolds	Genome size (bp)	G+C content (%)	Accession no.	GLM-based DDH estimate (Probability that the value exceeds 70%)^b^			
						**1**	**2**	**3**	**4**
*Streptomyces* sp. TP-A0882 (NBRC 110030)^a^ (**1**)	723.0	34	8,106,535	72.5	BBOK01000000	—	94.4% (97.1%)	45.1% (8.4%)	43.2% (5.8%)
*S. diastaticus* subsp. *ardesiacus* NBRC 15402^T^ (**2**)	1005.0	32	7,851,547	72.7	BEWC01000000		—	45.4% (8.8%)	43.2% (5.7%)
*S. coelicoflavus* NBRC 15399^T^ (**3**)	645.8	41	8,727,276	72.2	BEWB01000000			—	45.7% (9.5%)
*S. rubrogriseus* NBRC 15455^T^ (**4**)	896.2	21	8,454,317	72.2	BEWD01000000				—

^a^Data from our previous study^12^.

^b^Distances are inferred using Formula 2 (identities/high-scoring segment pair (HSP) length) from the set of HSPs representing the most unique matches obtained by comparing each pair of genomes. These distances are transformed into values analogous to the DDH using a generalized linear model inferred from an empirical reference dataset comprising real DDH values and genome sequences.

### NRPS and PKS gene clusters

In our previous study, we sequenced the genome of *Streptomyces* sp. TP-A0882 and identified BGCs for butyrolactol and thiazostatin^12^. The genome contains at least 14 gene clusters coding for proteins involved in NRPS and PKS pathways (Table 2). To validate whether taxonomically close strains share similar secondary metabolite biosynthetic pathways, in the current study we surveyed the NRPS and PKS gene clusters in the genomes of *S. diastaticus* subsp. *ardesiacus* NBRC 15402^T^, *S. coelicoflavus* NBRC 15399^T^, and *S. rubrogriseus* NBRC 15455^T^.

**Table 2:** Open reading frames (ORFs) encoding nonribosomal peptide synthetases (NRPSs) and polyketide synthases (PKSs) in NRPS and PKS gene clusters from *Streptomyces* sp. TP-A0882 (NBRC 110030).

Gene cluster	Presumed product	ORF (accession)^a^	Size (aa)	Domain organization
*nrps-1*	coelibactin	12–265 (WP_055468803)^c^	554	A(dhb)
		12–266 (WP_055468804)	2,246	T-C/A/T-C/A(cys)/T
		12–267 (WP_055468805)	1,857	C/A(cys)/MT/T-TE
*nrps-2*	coelichelin	12–104 (WP_055468733)	3,644	A(orn)/T/E-C/A(thr)/T/E-C/A(orn)/T
*nrps-3*	mCys-Val-…-x-Ser	*13–1 (in BBOK01000009)* ^b^	>*2,354*	*C/A(cys)/MT/T-C/A(val)…*
		*22–1 (in BBOK01000019)* ^b^	>*2,560*	*…E-C/A/T-C/A(ser)/T*
*nrps-4*	thiazostatin	2–333 (WP_055468178)	1,829	C/A(cys)/MT/T-TE
		2–328 (WP_055468176)	1,523	T-C/A(cys)/T
		2–326 (WP_053639878)	532	A(dhb)
*pks/nrps-1*	x-Val-Pro-pk	10–54 (WP_055469571)	1,303	C/A/T-TE
		10–53 (WP_063788334)	3,113	A(val)/T-C/A(pro)/T-KS/KR/ACP-TE
*t1pks-1*	butyrolactol	10–11 (WP_055469545)^c^	6,065	AT/ACP-KS/AT(mm)/DH/ER/KR/ACP-KS/AT(m)/KR/ACP-KS/AT(m)/DH/KR/ACP
		10–14 (WP_055469666)	2,083	KS/AT(m)/DH/ER/KR/ACP
		10–15 (WP_055469548)	3,365	KS/AT(m)/DH/KR/ACP-KS/AT(m)/DH/KR/ACP
		10–16 (WP_055469549)	3,462	KS/AT/DH/ER/KR/ACP-KS/KR/ACP
		10–17 (WP_055469550)	3,135	KS/AT(m)/KR/ACP-KS/AT(m)/KR/ACP
		10–18 (WP_055469551)	1,169	KS/DH/KR/ACP
*t1pks-2*	AHBA-diketide	2–307 (WP_055468168)	2,191	CoL(AHBA)/KR/ACP-KS/AT(m)/ACP
		2–306 (WP_055468167)	1,296	KS/AT(m)/ACP-TE
*t1pks-3*	unknown	18–62 (WP_063788240)	128	ACP
		18–61 (WP_055468074)	2,027	KS/AT(m)/DH/ER/KR/ACP
		18–60 (WP_051849763)	482	KS
*other t1pks(s)*	unknown(s)	*26–1 (WP_055470054)*	>*1,045*	*…AT(m)/DH/KR/ACP*
		26–2 (WP_055470053)	1,715	KS/AT(mm)/KR/ACP
		*26–3 (in BBOK01000023)* ^b^	>*2,325*	*KS/AT(m)/DH/KR/ACP-KS…*
		*13–248 (WP_055468920)*	>*354*	*…DH/KR/ACP*
		13–247 (WP_055468919)	3,111	KS/AT(m)/DH/KR/ACP-KS/AT(m)/ACP-TE
		13–232 (WP_055468914)	5,409	ACP-KS/AT(m)/DH/KR/ACP-KS/AT(m)/DH/KR/ACP-KS/AT(m)/DH/KR/ACP
		13–231 (WP_055468913)	1,862	KS/AT(m)/DH/KR/ACP
*t2pks-1*	gray spore pigment	7–97 (WP_030402764)	423	KS
		7–98 (WP_053637533)	422	KS
		7–99 (WP_030402766)	89	ACP
*t2pks-2*	kinamycin-like	15–178 (WP_031082067)	423	KS
		15–179 (WP_031184969)	407	KS
		15–180 (WP_030402549)	89	ACP
*t3pks-1*	THN	4–414 (WP_031081839)	374	KS
*t3pks-2*	phenolic lipid	7–128 (WP_037824347)	390	KS
*t3pks-3*	unknown	4–314 (WP_030398736)	361	KS

Abbreviations: A, adenylation; ACP, acyl carrier protein; AHBA, aminohydroxybenzoic acid; AMT, aminotransferase; AT, acyltransferase; C, condensation; CoL, CoA ligase; DH, dehydratase; E, epimerization; ER, enoylreductase; F, formyltransferase; KR, ketoreductase; KS, ketosynthase; m, malonyl-CoA: mCys, methyl-cysteine; mGly, methyl-glycine; mm, methylmalonyl-CoA; MT, methyltransferase; pk, moiety derived from PKS pathway; T, thiolation; TD, termination; TE, thioesterase; THN, tetrahydroxynaphthalene; x, unidentified amino-acid; y, unknown building block because A domain is not present in the module. Predicted substrates of A, AT, and CoL domains are shown in brackets.

^a^ORFs are shown as a combination of scaffold number and ORF number. Incompletely sequenced ORFs are shown in italics, and undetermined domains are shown as “…”.

^b^Because the ORFs are not registered in GenBank, accession numbers for the DNA sequences encoding each ORF are instead indicated in brackets.

^c^Encoded on the complementary strand.

*S. diastaticus* subsp. *ardesiacus* NBRC 15402^T^ harbors four NRPS gene (*nrps*) clusters, one hybrid PKS/NRPS gene (*pks/nrps*) cluster, at least four type I PKS gene (*t1pks*) clusters, two type II PKS gene (*t2pks*) clusters, and three type III PKS gene (*t3pks*) clusters, as shown in Tables 3 and 4. The number and types of gene clusters are same as those of *Streptomyces* sp. TP-A0882 and the sequences show >99% amino acid sequence identity to those of *Streptomyces* sp. TP-A0882 (NBRC 110030) based on BLAST analysis in all cases except ORF77-1 and ORF80-1 (Table 4). The structures of predicted products of the gene clusters from NBRC 15402^T^ also matched those of TP-A0882. These results suggested that the two *S. diastaticus* subsp. *ardesiacus* strains contain identical NRPS and PKS pathways.

**Table 3:** Numbers of secondary metabolite-biosynthetic gene clusters (smBGCs) encoded in each genome.

smBGC for	*S. diastaticus* subsp. *ardesiacus*		*S. coelicoflavus* NBRC 15399^T^	*S. rubrogriseus* NBRC 15455^T^
	TP-A0882	NBRC 15402^T^		
nonribosomal peptide (NRP)	4	4	4	4
hybrid polyketide (PK)/NRP	1	1	2	1
PK, type-I	>4^a^	>4	—^b^	>3
PK, type-II	2	2	3	2
PK, type-III	3	3	1	2
subtotal	>14	>14	10	>12
bacteriocin	1	1	1	1
butyrolactone	—	—	1	1
ectoine	1	1	1	1
indole	1	1	1	1
lantipeptide	1	—	3	2
melanin	1	1	1	1
oligosaccharide	1	1	—	—
siderophore, non-NRP	2	2	2	3
terpene	6	6	6	5
others	—	—	2	—
subtotal	14	13	18	15
total	>28	27	28	27

^a^As some type-I PKS gene clusters were not completely sequenced, exact numbers are unclear.

^b^Not detected.

**Table 4:** ORFs encoding NRPSs and PKSs in NRPS and PKS gene clusters from *S*. *diastaticus* subsp. *ardesiacus* NBRC 15402^T^.

Gene cluster	Presumed product	ORF^a^	Size (aa)	Domain organization	Closest homolog (accession, origin, % of identity/similarity)^b^
*nrps-1*	coelibactin	1–1240^c^	542	A(dhb)	WP_055468803, *Streptomyces* sp. NBRC 110030, 97/97
		1–1241	2,213	T-C/A/T-C/A(cys)/T	KOX46963, *Streptomyces* sp. NRRL F-7442, 99/99 (WP_055468804, *Streptomyces* sp. NBRC 110030, 99/99)
		1–1242	1,857	C/A(cys)/MT/T-TE	WP_053663986, *Streptomyces* sp. NRRL F-7442, 99/99 (WP_055468805, *Streptomyces* sp. NBRC 110030, 99/99)
*nrps-2*	coelichelin	1–1084	3,644	A(orn)/T/E-C/A(thr)/T/E-C/A(orn)/T	KOX26695, *Streptomyces* sp. NRRL F-4707, 99/99 (WP_055468733, *Streptomyces* sp. NBRC 110030, 99/99)
*nrps-3*	mCys-Val-x-x-Ser	1–84	3,616	C/A(cys)/MT/T-C/A(val)/T/E-C	WP_051908973, *Streptomyces* sp. NRRL F-5635, 99/99^d^
		1–85	3,241	A/T/E-C/A/T-C/A(ser)/T	EHN79578, *Streptomyces coelicoflavus* ZG0656, 93/94^d^
*nrps-4*	thiazostatin	10–229	1,829	C/A(cys)/MT/T-TE	WP_031081050, *Streptomyces* sp. NRRL S-1831, 99/99 (WP_055468178, *Streptomyces* sp. NBRC 110030, 99/99)
		10–234	1,535	T-C/A(cys)/T	WP_031184402, *Streptomyces* sp. NRRL F-5635, 99/98 (WP_055468176, *Streptomyces* sp. NBRC 110030, 98/98)
		10–236	532	A(dhb)	WP_053639878, *Streptomyces* sp. NBRC 110030, 99/99
*pks/nrps-1*	x-Val-Pro-pk	5–41	1,303	C/A/T-TE	WP_055469571, *Streptomyces* sp. NBRC 110030, 99/99
		5–40	3,105	A(val)/T-C/A(pro)/T-KS/KR/ACP-TE	WP_063788334, *Streptomyces* sp. NBRC 110030, 99/99
*t1pks-1*	butyrolactol	43–30^c^	6,062	AT/ACP-KS/AT(mm)/DH/ER/KR/ACP-KS/AT(m)/KR/ACP-KS/AT(m)/DH/KR/ACP	WP_055469545, *Streptomyces* sp. NBRC 110030, 99/99
		*43–33*	>398	*KS…*	WP_055469666, *Streptomyces* sp. NBRC 110030, 98/98
		*58–1*	>*1,655*	*…AT/DH/ER/KR/ACP*	WP_055469666, *Streptomyces* sp. NBRC 110030, 98/98
		*58–2*	>426	*KS…*	WP_055469548, *Streptomyces* sp. NBRC 110030, 99/99
		*62–1*	>*1,601*	*…AT(m)/DH/KR/ACP-KS…*	WP_055469548, *Streptomyces* sp. NBRC 110030, 97/98
		*5–1*	>*1,334*	*…AT(m)/DH/KR/ACP*	WP_055469548, *Streptomyces* sp. NBRC 110030, 97/98
		5-2	3,464	KS/AT/DH/ER/KR/ACP-KS/KR/ACP	WP_055469549, *Streptomyces* sp. NBRC 110030, 99/99
		5-3	3,141	KS/AT(m)/DH/KR/ACP-KS/AT(m)/KR/ACP	KOT98773, *Streptomyces* sp. NRRL F-4711, 99/99 (WP_055469550, *Streptomyces* sp. NBRC 110030, 99/99)
		5-4	1,169	KS/DH/KR/ACP	KOT98774, *Streptomyces* sp. NRRL F-4711, 99/99 (WP_055469551, *Streptomyces* sp. NBRC 110030, 99/99)
*t1pks-2*	AHBA-diketide	10–255	2,191	CoL(AHBA)/KR/ACP-KS/AT(m)/ACP	WP_051908920, *Streptomyces* sp. NRRL F-5635, 99/99 (WP_055468168, *Streptomyces* sp. NBRC 110030, 99/99)
		10–256	1,296	KS/AT(m)/ACP-TE	WP_053663292, *Streptomyces* sp. NRRL F-7442, 99/99 (WP_055468167, *Streptomyces* sp. NBRC 110030, 99/99)
*t1pks-3*	unknown	6–621	128	ACP	WP_063788240, *Streptomyces* sp. NBRC 110030, 99/100
		6–622	2,027	KS/AT(m)/DH/ER/KR/ACP	KOX28560, *Streptomyces* sp. NRRL F-4707, 99/99 (WP_055468074, *Streptomyces* sp. NBRC 110030, 99/100)
		6–623	482	KS	WP_051783751, *Streptomyces* sp. NRRL F-5555, 99/99 (WP_051849763, *Streptomyces* sp. NBRC 110030, 99/99)
*other t1pks(s)*	unknown(s)	*53-3*	>*1,507*	*…AT(m)/DH/KR/ACP*	WP_055470054, *Streptomyces* sp. NBRC 110030, 99/98
		53-2	1,650	KS/AT(mm)/KR/ACP	KOX41189, *Streptomyces* sp. NRRL F-7442, 99/99 (WP_055470053, *Streptomyces* sp. NBRC 110030, 99/99)
		*53-1*	>*2,463*	*KS/AT(m)/DH/KR/ACP-KS/AT…*	WP_033305239, *Streptomyces atroolivaceus*, 57/67^d^
		*77-1*	>*762*	*…KR/ACP-KS…*	AHH99923, *Kutzneria albida* DSM 43870, 63/74
		*80-1*	>*489*	*…KS…*	WP_040741646, *Nocardia tenerifensis*, 70/78
		*59-1*	>*350*	*…KR/ACP*	WP_055468920, *Streptomyces* sp. NBRC 110030, 100/100
		*59-2*	>*1,370*	*KS/AT(m)/DH…*	WP_055468919, *Streptomyces* sp. NBRC 110030, 99/99
		*20-1*	>*1,605*	*…KR/ACP-KS/AT(m)/ACP-TE*	WP_055468919, *Streptomyces* sp. NBRC 110030, 99/99
		20-16	5,412	ACP-KS/AT(m)/DH/KR/ACP-KS/AT(m)/DH/KR/ACP-KS/AT(m)/DH/KR/ACP	WP_053639270, *Streptomyces* sp. NRRL F-4707, 99/99 (WP_055468914, *Streptomyces* sp. NBRC 110030, 99/99)
		20-17	1,862	KS/AT(m)/DH/KR/ACP	WP_053639271, *Streptomyces* sp. NRRL F-4707, 99/99 (WP_055468913, *Streptomyces* sp. NBRC 110030, 99/99)
*t2pks-1*	gray spore pigment	2–814	423	KS	WP_030402764, *Streptomyces* sp. NBRC 110030, 100/100
		2–813	422	KS	WP_053637533, *Streptomyces* sp. NBRC 110030, 99/100
		2–812	89	ACP	WP_030402766, *Streptomyces* sp. NBRC 110030, 100/100
*t2pks-2*	kinamycin-like	15–126	423	KS	KOX34713, *Streptomyces* sp. NRRL F-4707, 100/100 (WP_031082067, *Streptomyces* sp. NBRC 110030, 99/100)
		15–127	407	KS	WP_031184969, *Streptomyces* sp. NRRL F-5635, 99/100 (WP_055468989, *Streptomyces* sp. NBRC 110030, 99/99)
		15–128	89	ACP	WP_030402549, *Streptomyces* sp. NBRC 110030, 99/100
*t3pks-1*	THN	8–149	374	KS	WP_031081839, *Streptomyces* sp. NBRC 110030, 100/100
*t3pks-2*	phenolic lipid	1–740	390	KS	WP_037824347, *Streptomyces* sp. NBRC 110030, 99/99
*t3pks-3*	unknown	10–248	361	KS	WP_030398736, *Streptomyces* sp. NBRC 110030, 100/100

Abbreviations are the same as those of Table 2.

^a^ORFs are shown as a combination of scaffold number and ORF number. Incompletely sequenced ORFs are shown in italics, and undetermined domains are shown as “…”.

^b^Parentheses indicate that the closest homolog is not from *Streptomyces* sp. TP-A0882 (NBRC 110030).

^c^Encoded on the complementary strand.

^d^Although homologs in *Streptomyces* sp. TP-A0882 did not appear as high score hits in basic local alignment search tool analyses because they are not registered in GenBank, they are present in scaffolds 13 (BBOK01000009), 22 (BBOK01000019), and 26 (BBOK01000023) of the *Streptomyces* sp. TP-A0882 genome.

*S. coelicoflavus* NBRC 15399^T^ harbors four *nrps* clusters, two *pks/nrps* clusters, three *t2pks* clusters, and one *t3pks* cluster, as shown in Table 5. Unlike typical *Streptomyces* strains, *t1pks* cluster is not present in this strain. *nrps-i*, *nrps-ii*, *pks/nrps-i*, *t2pks-i*, and *t3pks-i* were predicted to be responsible for the synthesis of coelibactin, coelichelin, prodiginine, gray spore pigment, and tetrahydroxynaphthalene (THN), respectively, based on high similarities (85–99% amino acid sequence identity) to SCO7681-7683, SCO0492 (CchH), SCO5886-SCO5894 (Red), SCO5318-SCO5316 (WhiE), and SCO1206 (RppA) of *Streptomyces coelicolor* A3(2)^6,13^, respectively. Based on the domain and module organizations and substrate selective residues in the A domains, *nrps-iii* and *nrps-iv* were predicted to synthesize nonribosomal peptides consisting of eight amino acids and 13 amino acids, respectively. The product of *pks/nrps-ii* was speculated to be a novel oxazolomycin analog because the domain organization is similar, but not identical, to that of the BGCs for oxazolomycins^14^. Although the remaining two gene clusters (*t2pks-ii*, *t2pks-iii*) are likely to be responsible for the synthesis of aromatic polyketides, the structures were not predicted from the sequence information alone. Analysis of the genome sequence of *S. coelicoflavus* strain ZG0656, the only *S. coelicoflavus* strain of which genome sequence is published^15^, indicated that all of the *S. coelicoflavus* NBRC 15399^T^ gene clusters (Table 5) are present also in strain ZG0656 with >97% amino acid sequence identity based on BLAST comparisons.

**Table 5:** ORFs encoding NRPSs and PKSs in NRPS and PKS gene clusters of *S. coelicoflavus* NBRC 15399^T^.

Gene cluster	Presumed product	ORF^a^	Size (aa)	Domain organization	Closest homolog (accession, origin, % of identity/similarity)^b^
*nrps-i*	coelibactin	3–140^c^	554	A(dhb)	EHN75391, *S. coelicoflavus* ZG0656, 99/99 (CAC17498, *S. coelicolor* A3(2), 85/89)
		3–141	2,250	T-C/A/T-C/A(cys)/T	EHN75408, *S. coelicoflavus* ZG0656, 99/99 (CAC17499, *S. coelicolor* A3(2), 86/89)
		3–142	1,857	C/A(cys)/MT/T-TE	EHN75409, *S. coelicoflavus* ZG0656, 99/99 (CAC17500, *S. coelicolor* A3(2), 89/91)
*nrps-i*	coelichelin	6–362	3,666	A(orn)/T/E-C/A(thr)/T/E-C/A(orn)/T	KPC76200, *Streptomyces* sp. NRRL WC-3753, 99/99 (EHN78004, *S. coelicoflavus* ZG0656, 99/98; CAB53322, *S. coelicolor* A3(2), 86/90)
*nrps-iii*	x-x-Ser-mCys-Val-x-x-Ser	3–549	3,637	C/A(cys)/MT/T-C/A(val)/T/E-C/A/T/E-C/A/T-C/A(ser)/T	EHN75118, *S. coelicoflavus* ZG0656, 99/99
		3–550	3,271	A/T/E-C/A/T-C/A(ser)/T	EHN79578, *S. coelicoflavus* ZG0656, 99/98
*nrps-iv*	Gly-y-Asp-Tyl-Thr-x-Asp-Gly-Pro-Gly-Gly-Ala-mGly	2–543	6,937	C/A(gly)/T-C/T-C/A(asp)/T/E-C/A(tyl)/T-C/A(thr)/T-C/A/T/E	KPC71694, *Streptomyces* sp. NRRL WC-3753, 99/99 (EHN72150, *S. coelicoflavus* ZG0656, 99/99)
		2–544	4,213	C/A(asp)/T-C/A(gly)/T-C/A(pro)/T-C/A(gly)/T	KPC71705, *Streptomyces* sp. NRRL WC-3753, 99/99 (EHN72136, *S. coelicoflavus* ZG0656, 99/99)
		2–545	3,865	C/A(gly)/T-C/A(ala)/T-C/A(gly)/MT/T-TE	WP_054100963, *Streptomyces* sp. NRRL WC-3753, 99/99 (EHN72117, *S. coelicoflavus* ZG0656, 99/99)
*pks/nrps-i*	prodiginine	3–99	1,012	KS/KS	KPC87173, *Streptomyces* sp. NRRL WC-3753, 99/99 (EHN75481, *S. coelicoflavus* ZG0656, 99/99; CAA16487, *S. coelicolor* A3(2), 91/94)
		3–107^c^	407	KS	EHN75487, *S. coelicoflavus* ZG0656, 100/100 (CAA16177, *S. coelicolor* A3(2), 94/97)
		3–108^c^	81	ACP	EHN75488, *S. coelicoflavus* ZG0656, 100/100 (CAA16178, *S. coelicolor* A3(2), 96/97)
		3–110	87	ACP	EHN77254, *S. coelicoflavus* ZG0656, 100/100 (CAA16180, *S. coelicolor* A3(2), 95/95)
		3–111	636	ACP/AMT	KPC87185, *Streptomyces* sp. NRRL WC-3753, 99/98 (EHN75478, *S. coelicoflavus* ZG0656, 98/98; CAA16181, *S. coelicolor* A3(2), 88/89)
		3–112	532	A(cys)	KPC87186, *Streptomyces* sp. NRRL WC-3753, 99/99 (EHN75480, *S. coelicoflavus* ZG0656, 99/99; CAA16182, *S. coelicolor* A3(2), 93/95)
		3–113	2,306	CoL(NH2)/T-KS/AT(m)/ACP/AMT	EHN77210, *S. coelicoflavus* ZG0656, 99/99 (CAA16183, *S. coelicolor* A3(2), 87/90)
		3–115	280	TE	EHN75475, *S. coelicoflavus* ZG0656, 99/99 (CAA16185, *S. coelicolor* A3(2), 96/96)
*pks/nrps-ii*	oxazolomycin-like	7–245^c^	842	ACP-TD	WP_051005867, *S. coelicoflavus* ZG0656, 98/98
		7–244^c^	1,752	KS/ACP-C/FkbH	WP_054101954, *Streptomyces* sp. NRRL WC-3753, 99/98 (WP_051005868, *S. coelicoflavus* ZG0656, 98/98)
		7–242	2,968	DH/ACP/ACP/ACP/DH-KS/KR/ACP-KS/ACP	WP_054101951, *Streptomyces* sp. NRRL WC-3753, 97/97 (EHN75054, *S. coelicoflavus* ZG0656, 97/97)
		7–241	3,008	C/A(ser)/T-C/A/MT/T-C	KPC72343, *Streptomyces* sp. NRRL WC-3753, 99/99 (EHN75030, *S. coelicoflavus* ZG0656, 99/99)
		7–237	4,903	KS/DH/KR/ACP-KS/DH/KR/ACP-KS/DH/KR/MT/ACP	KPC72421, *Streptomyces* sp. NRRL WC-3753, 98/98 (EHN75036, *S. coelicoflavus* ZG0656, 97/97)
		7–236	1,158	F/A(gly)/T	KPC71002, *Streptomyces* sp. NRRL WC-3753, 99/99 (EHN77489, *S. coelicoflavus* ZG0656, 99/99)
		7–234	879	KS/ACP	KPC71004, *Streptomyces* sp. NRRL WC-3753, 99/99 (EHN75023, *S. coelicoflavus* ZG0656, 99/99)
		7–233	6,079	KS/KR/MT/ACP-C/A(gly)/T-KS/DH/KR/ACP-KS/KR/ACP-KS	WP_054102642, *Streptomyces* sp. NRRL WC-3753, 98/98 (EHN78704, *S. coelicoflavus* ZG0656, 98/98)
		7–232	1,106	AT/AT(m)	EHN78700, *S. coelicoflavus* ZG0656, 99/99
*t2pks-i*	gray spore pigment	11–215	423	KS	KPC88984, *Streptomyces* sp. NRRL WC-3753, 100/100 (EHN75824, *S. coelicoflavus* ZG0656, 99/99; CAB45606, *S. coelicolor* A3(2), 98/99)
		11–214	424	KS	KPC88985, *Streptomyces* sp. NRRL WC-3753, 99/99 (EHN75823, *S. coelicoflavus* ZG0656, 98/98; CAB45607, *S. coelicolor* A3(2), 98/98)
		11–213	89	ACP	EHN75822, *S. coelicoflavus* ZG0656, 100/100 (CAB45608, *S. coelicolor* A3(2), 98/98)
*t2pks-ii*	unknown	1–30	84	ACP	EHN79053, *S. coelicoflavus* ZG0656, 100/100
		1–31	422	KS	EHN79055, *S. coelicoflavus* ZG0656, 100/100
		1–32	416	KS	EHN79056, *S. coelicoflavus* ZG0656, 99/99
*t2pks-iii*	unknown	14–63	421	KS	EHN77732, *S. coelicoflavus* ZG0656, 100/100
		14–62	415	KS	KPC71304, *Streptomyces* sp. NRRL WC-3753, 99/99 (EHN77731, *S. coelicoflavus* ZG0656, 99/99)
*t3pks-i*	THN	5–164	374	KS	EHN79529, *S. coelicoflavus* ZG0656, 100/100 (CAC01488, *S. coelicolor* A3(2), 91/95)

Abbreviations are the same as those of Table 2.

^a^ORFs are shown as a combination of scaffold number and ORF number.

^b^If the homolog in *S. coelicoflavus* ZG0656 is not the closest and/or *Streptomyces coelicolor* A3(2) harbors the homolog, it is shown in parentheses.

^c^Encoded on the complementary strand.

*S. rubrogriseus* NBRC 15455^T^ harbors four *nrps* clusters, one *pks/nrps* cluster, at least three *t1pks* clusters, two *t2pks* clusters, and two *t3pks* clusters (Table 6). *nrps-a*, *nrps-b*, *nrps-c*, *pks/nrps-a*, *t1pks-a*, *t1pks-b*, *t2pks-a*, *t3pks-a*, and *t3pks-b* were predicted to be responsible for the synthesis of coelibactin, coelichelin, calcium-dependent antibiotic (CDA), prodiginine, coelimycin, eicosapentaenoic acid, gray spore pigment, THN, and phenolic acid, respectively, based on high similarities (91–100% amino acid sequence identities) to SCO7681-7683, SCO0492 (CchH), SCO3230-SCO3032 (CDA peptide synthetases), SCO5886-SCO5894 (Red), SCO6275-SCO6273 (Cpk), SCO0126-SCO0127, SCO5318-SCO5316 (WhiE), SCO1206 (RppA), and SCO7671 (SrsA ortholog)^6,13^, respectively. Based on the domain and module organization and substrate selective residues in the A domains, *nrps-d* was predicted to synthesize a peptide containing cysteine. Other *t1pks* cluster(s) were not completely sequenced, but their predicted PKS proteins do not have high sequence similarity to the known PKS proteins, suggesting that the product(s) might be novel. *t2pks-b* is likely to synthesize aromatic polyketides, but the products could not be predicted because the sequence does not show a high degree of similarity to any PKS whose products have been elucidated. Among the 12 gene clusters, all except the other *t1pks* genes and *t2pks-b* show >93% sequence similarity to the corresponding genes from *S. coelicolor* A3(2), suggesting that most of the gene clusters in *S. rubrogriseus* NBRC 15455^T^ are present also in *S. coelicolor* A3(2).

**Table 6:** ORFs encoding NRPSs and PKSs in NRPS and PKS gene clusters of *S. rubrogriseus* NBRC 15455^T^.

Gene cluster	Presumed product	ORF^a^	Size (aa)	Domain organization	Closest homolog (accession, origin, % of identity/similarity)^b^
*nrps-a*	coelibactin	7–361^c^	553	A(dhb)	CAC17498, *S. coelicolor* A3(2), 98/98
		7–362	2,240	T-C/A/T-C/A(cys)/T	CAC17499, *S. coelicolor* A3(2), 96/97
		7–363	1,842	C/A(cys)/MT/T-TE	CAC17500, *S. coelicolor* A3(2), 97/97
*nrps-b*	coelichelin	4–1115	3,649	A(orn)/T/E-C/A(thr)/T/E-C/A(orn)/T	CAB53322, *S. coelicolor* A3(2), 95/96
*nrps-c*	CDA	13–202	7,395	C/A(ser)/T-C/A(thr)/T-C/A(trp)/T/E-C/A(asp)/T-C/A(asp)/T-C/A(hpg)/T/E	CAB38518, *S. coelicolor* A3(2), 95/96
		13–203	3,658	C/A(asp)/T-C/A(gly)/T-C/A(asn)/T/E	CAB38517, *S. coelicolor* A3(2), 96/97
		13–204	2,429	C/A/T-C/A(trp)/T-TE	CAD55498, *S. coelicolor* A3(2), 97/97
*nrps-d*	x-y-Cys	1–88	1,177	A/T-C/T	SDT78734, *Streptomyces* sp. 2114.2, 98/98 (CAA18918, *S. coelicolor* A3(2), 98/98)
		1–89	1,413	C/A(cys)/T-TE	CAA18919, *S. coelicolor* A3(2), 96/96
*pks/nrps-a*	prodiginine	3–389	932	KS/KS	CAA16487, *S. coelicolor* A3(2), 96/96
		3–381^c^	407	KS	CAA16177, *S. coelicolor* A3(2), 99/99
		3–380^c^	81	ACP	CAA16178, *S. coelicolor* A3(2), 99/100
		3–378	87	ACP	CAA16180, *S. coelicolor* A3(2), 100/100
		3–377	641	ACP/ACP/AMT	CAA16181, *S. coelicolor* A3(2), 97/97
		3–376	532	A(cys)	CAA16182, *S. coelicolor* A3(2), 98/99
		3–375	2,298	CoL/T-KS/AT(m)/ACP/AMT	SDT77027, *Streptomyces* sp. 2114.2, 96/96 (CAA16183, *S. coelicolor* A3(2), 95/96)
		3–373	280	TE	CAA16185, *S. coelicolor* A3(2), 98/100
*t1pks-a*	coelimycin	1–2	4,563	KS/AT(m)/ACP-KS/AT(m)/DH/KR/ACP-KS/AT(m)/DH/KR/ACP-	SDT78409, *Streptomyces* sp. 2114.2, 93/95 (CAD55506, *S. coelicolor* A3(2), 98/98)
		*1-1*	>*595*	*KS…*	CAC22145, *S. coelicolor* A3(2), 94/97
		*34-1*	>*1,743*	*…AT/DH/KR/ACP-KS…*	CAC22145, *S. coelicolor* A3(2), 92/95
		*36-1*	>*907*	*…DH/KR/ACP*	CAC22145, *S. coelicolor* A3(2), 91/93
		*36-2*	>*582*	*KS…*	CAC22144, *S. coelicolor* A3(2), 96/97
*t1pks-b*	EPA	7–477	2,074	KS/AT(m)/ACP/KR/DH	SDS27436, *Streptomyces* sp. 2114.2, 96/96 (CAB52353, *S. coelicolor* A3(2), 95/95)
		7–476	2,240	KS/AT	CAB52354, *S. coelicolor* A3(2), 96/97
*other t1pks(s)*	unknown(s)	*6-1*	>*1,561*	*KS/AT/ACP-KS…*	SCE45938, *Streptomyces* sp. DvalAA-14, 74/80
		*35-1*	>*933*	*…DH/KR/ACP*	APD71595, *Streptomyces* sp. MM3, 54/65
		35-2	1,622	KS/AT(mm)/KR/ACP	AJC56296, *Streptomyces* sp. 769, 52/64
		*35-3*	>*493*	*KS…*	APD71977, *Streptomyces* sp. MM3, 69/81
		*9–576*	>*1,388*	*…AT(mm)/KR/ACP-TE*	SCD97877, *Streptomyces* sp. DvalAA-14, 66/75
		9–571^c^	693	KS/ACP	WP_052397599, *Streptomyces* sp. NRRL F-5123, 75/81
		9–569^c^	3,992	KS/ACP-KS/AT(mm)/DH/KR/ACP-KS/AT(m)/DH/KR/ACP	CDR05500, *Streptomyces iranensis*, 48/58
*t2pks-a*	gray spore pigment	10–372	423	KS	CAB45606, *S. coelicolor* A3(2), 98/99
		10–371	424	KS	CAB45607, *S. coelicolor* A3(2), 99/99
		10–370	90	ACP	CAB45608, *S. coelicolor* A3(2), 100/100
*t2pks-b*	unknown	9–560	82	ACP	WP_031518191, *Streptomyces* sp. NRRL F-5123, 81/89
		9–561	421	KS	WP_031518190, *Streptomyces* sp. NRRL F-5123, 85/91
		9–562	421	KS	WP_033177057, *Streptomyces* sp. URHA0041, 86/91
*t3pks-a*	THN	4–335	374	KS	SDS82518, *Streptomyces* sp. 2114.2, 96/98 (CAC01488, *S. coelicolor* A3(2), 96/98)
*t3pks-b*	phenolic acid	7–351	391	KS	CAC17488, *S. coelicolo*r A3(2), 90/91

CDA, calcium-dependent antibiotic; EPA, eicosapentaenoic acid. The other abbreviations are the same as those of Table 2.

^a^ORFs are shown as a combination of scaffold number and ORF number. Incompletely sequenced ORFs are shown in italics, and undetermined domains are shown as “…”.

^b^Parentheses indicate that the closest homolog is not from *S. coelicolor* A3(2).

^c^Encoded on the complementary strand.

### Conservation of NRPS and PKS gene clusters among taxonomically close species

**Figure 1:**
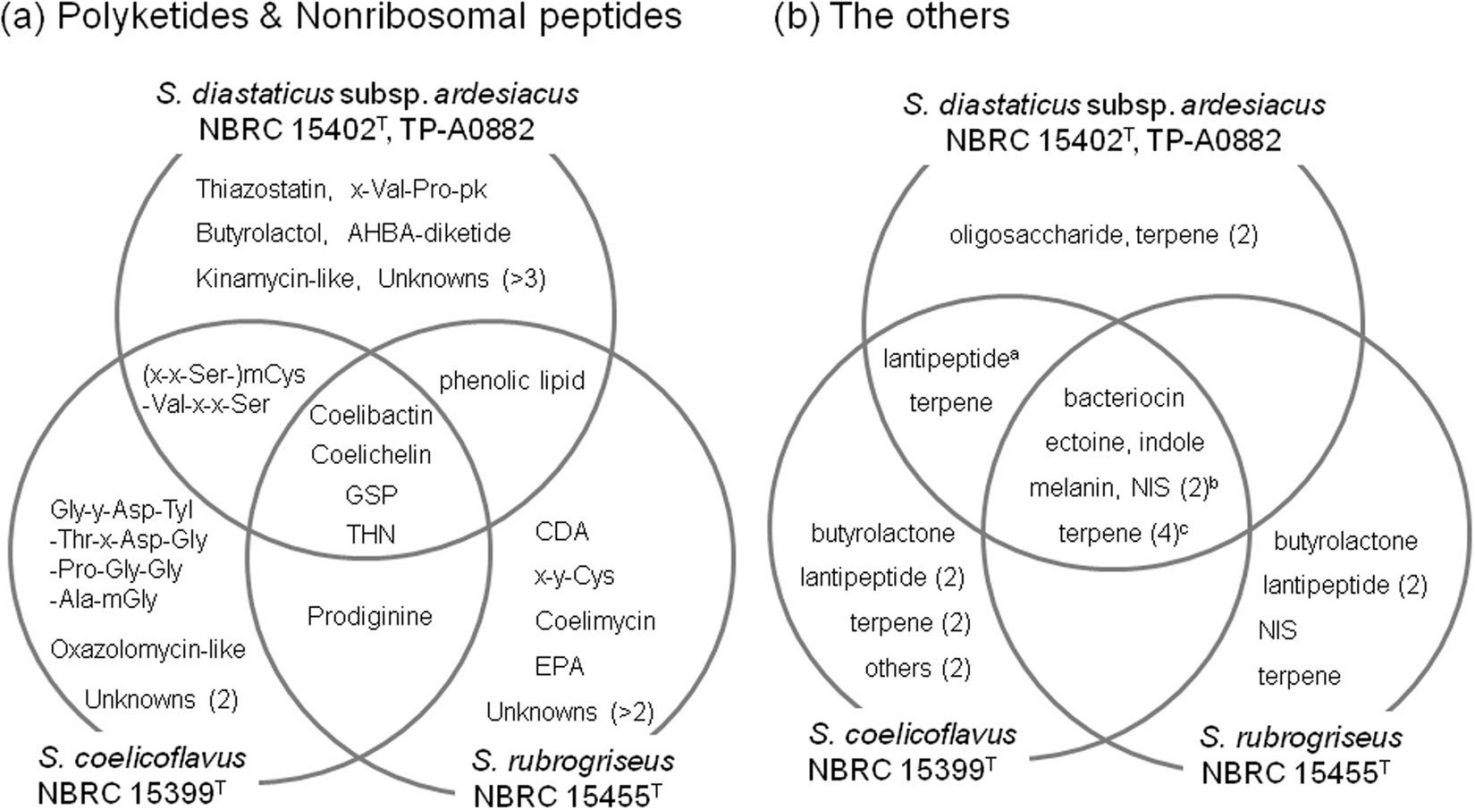
Schematic diagram showing diversity of NRPS & PKS gene clusters (**a**) and the other biosynthetic gene clusters (**b**) in the taxonomically close species. As *nrps-3* of the *S. diastaticus* subsp. *ardesiacus* strains and *nrps-iii* of *S. coelicoflavus* NBRC 15399^T^ show partial sequence similarity, the diagram shows putative sharing between these two species. However, the gene products of *nrps-3* and *nrps-iii* are divergent (mCys-Val-x-x-Ser and x-x-Ser-mCys-Val-x-x-Ser, respectively). Abbreviations: CDA, calcium-dependent antibiotic; EPA, eicosapentaenoic acid; GPS, gray spore pigment; m, methyl-; NIS, NRPS-independent siderophore; pk, moiety derived from PKS pathway; THN, tetrahydroxynaphthalene; x, unidentified amino-acid; y, unknown building block. ^a^The lantipeptide BGC, whose precursors peptide sequences are AVLINLDhbDDGCGDhaDhbCDhaDhaPCADhbNVA and CNGDhaCADhbNVA, is not present in the genome of of *S. diastaticus* subsp. *ardesiacus* NBRC 15402^T^; ^b^including desferrioxamine; ^c^albaflavenone, hopene, carotenoid & gosmin.

As summarized in Fig. 1a, BGCs for coelibactin, coelichelin, gray spore pigment, and THN are present in all of the strains. The prodiginine biosynthetic gene (*red*) cluster is not present in *S. diastaticus* subsp. *ardesiacus* strains NBRC 15402^T^ and TP-A0882, but is present in both *S. coelicoflavus* NBRC 15399^T^ and *S. rubrogriseus* NBRC 15455^T^. The phenolic lipid biosynthetic gene (*srs*) cluster is present in both *S. diastaticus* subsp. *ardesiacus* strains and *S. rubrogriseus* NBRC 15455^T^. Products of the *nrps-3* cluster from the *S. diastaticus* subsp. *ardesiacus* strains and the *nrps-iii* cluster from *S. coelicoflavus* NBRC 15399^T^ include mCys-Val-x-x-Ser. However, their products are actually not the same (*S. diastaticus* subsp. *ardesiacus* strains, mCys-Val-x-x-Ser; *S. coelicoflavus* NBRC 15399^T^, x-x-Ser-mCys-Val-x-x-Ser). Overall, the *S. diastaticus* subsp. *ardesiacus* strains, *S. coelicoflavus* NBRC 15399^T^, and *S. rubrogriseus* NBRC 15455^T^ harbor at least eight, four, and six species-specific gene clusters, respectively.

### The other secondary metabolite-biosynthetic gene clusters

In addition to NRPS and PKS gene clusters, the other smBGCs were also investigated. Thirteen to 18 gene clusters are encoded in each genome as shown in Table 3. Table 7 lists the clusters with putative products and loci. Homologous gene clusters are aligned in the same row in the table. *S. diastaticus* subsp. *ardesiacus* TP-A0882 and NBRC 15402^T^ shared the same set of gene clusters, except for a BGC for lantipeptides, suggesting that the two strains contain almost identical secondary metabolite biosynthetic pathways. Among the 18 BGCs of *S. coelicoflavus* NBRC 15399^T^, 13 are present also in *S. coelicoflavus* strain ZG0656 whereas three lantipeptide and two terpene BGCs are not. All 15 BGCs identified from *S. rubrogriseus* NBRC 15455^T^ are present also in *S. coelicolor* A3(2) (data not shown). BGCs for bacteriocin, ectoine, indole melanine, two siderophores, four terpenes are sheared among the three species, whereby 3 to 5 BGCs are specific in each species (Table 7, Fig. 1b).

**Table 7:** Loci encoding the other smBGCs in the draft genome sequences.

smBGC for	Putative product (Most similar known cluster)^a^	*S. diastaticus* subsp. *ardesiacus*		*S. coelicoflavus* NBRC 15399^T^	*S. rubrogriseus* NBRC 15455^T^
		TP-A0882	NBRC 15402^T^		
Bacteriocin	Informatipeptin	3,881–14,096, s07^b^	1,033,543–1,043,758, s01	109,209–119,424, s06	825,412–835,627, s04
Butyrolactone	unidentified^c^	ND^d^	ND	143,762–189,159, s14	ND
Butyrolactone	—^e^	ND	ND	ND	1–8,053, s03
Ectoine	Ectoine	299,195–309,593, s13	68,465–78,863, s18	229,038–239,436, s12	666,768–677,166, s06
Indole	unidentified	53,333–74,460, s16	737,576–758,703, s10	123,011–144,138, s13	127,502–148,620, s07
Lantipeptide	2 or 3 kinds of peptides^f^	337,776–362,051, s07	ND	33,290–58,341, s23	ND
Lantipeptide	GLVNLDhbDDNCGDhaDhbCGACDhbDhbNVA^g^	ND	ND	143,762–189,159, s14	ND
Lantipeptide	unidentified	ND	ND	582,852–607,263, s03	ND
Lantipeptide	DhbGDhaRADhaLLLCGDDhaDhaLDhaIDhbDhbCN^g^	ND	ND	ND	400,232–422,952, s01
Lantipeptide	AQFGEGDhbFDhbDhaPDhaDhaYAIGDhbRCPICC^g^	ND	ND	ND	1,370,541–1,405,635, s04
Melanin	Melanin	343,594–354,160,s02	476,879–487,445, s09	291,936–302,595, s01_2	359,033–369,602, s09
Oligosaccharide	unidentified	1–24,831, s10	225,676–260,720, s15	ND	ND
Siderophore	Desferrioxamine B	258,147–269,916, s02	561,212–572,981, s09	191,379–203,157, s01_2	266,567–278,345, s09
Siderophore	—	138,812–150,764, s15	57,784–69,736, s15	26,019–38,040, s03	549,038–560,963, s03
Siderophore	unidentified	ND	ND	ND	11,549–66,510, s03
Terpene	Albaflavenone	200,318–221,331,s01	778,230–799,243, s02	137,667–158,680, s11	301,202–322,287, s10
Terpene	Hopene	408,409–435,138, s11	560,033–586,762, s01	21,764–48,513, s07	495,633–522,374, s01
Terpene	Carotenoid	119,566–143,614, s16	668,873–692,929, s10	44,866–68,934, s13	44,374–68,462, s07
Terpene	Geosmin^h^	160,585–182,786, s09	190,986–213,187, s20	424,806–447,016, s03	207,587–229,767, s03
Terpene	unidentified	17,748–38,641, s19	161,086–181,979, s01	ND	ND
Terpene	—	241,318–265,214, s09	201,308–225,195, s05	ND	ND
Terpene	2–methylisoborneol	ND	ND	306,224–319,060, s13	ND
Terpene	Isorenieratene	ND	ND	110,932–136,512, s20	ND
Terpene	—	ND	ND	ND	1–20,497, s22
Other	Lomaiviticin	ND	ND	100,475–140,891, s14	ND
Other	unidentified	ND	ND	18,943–60,076, s18	ND

^a^When the outputs of antiSMASH showed >40% gene similarities, we putatively considered them as putative products; ^b^Locus is shown as start-end positions and scaffold no. (sxx means scaffold000xx); ^c^As analysis using antiSMASH output product names but the gene similarities were less 40% gene similarity, the products are shown as unidentified; ^d^Not detected; ^e^No output; ^f^AVLINLDhb(didehydrobutyrine)DDGCGDha(didehydroalanine)DhbCDhaDhaPCADhbNVA & CNGDhaCADhbNVA in *S. diastaticus* subsp. *ardesiacus* TP-A0882, DhaDGGCGDhaDhbCGNACIDhaDhaGDha, INLDhbDDGCGDhaDhbCDhaDhaPCADhbNVA & CKGDhaCADhbNVA in *S. coelicoflavus* NBRC 15399^T^; ^g^Core peptide amino acid sequence predicted by antiSMASH; ^h^based on the similarity to BGCs for giosmin.

## Discussion

Genome analysis conducted in this study shows that *S. diastaticus* subsp. *ardesiacus* strains TP-A0882 and NBRC 15402^T^ share an almost identical set of smBGCs, while *S. coelicoflavus* strains NBRC 15399^T^ and ZG0656 shared their own similar set of gene clusters. Previous studies on *Nocardia brasiliensis*^8^ and *Salinispora* species^16^ have also shown that most smBGCs are common within each species, with strain-specific ones being relatively limited. These results suggest that actinomycete strains belonging to the same species are also likely to possess similar secondary metabolite biosynthetic pathways.

In contrast, only a limited number of smBGC are shared by different species examined in this study, even though they have >99% 16S rRNA gene sequence similarity and are thus considered taxonomically close. We identified totally 49 different smBGCs including 25 NRPS and PKS gene clusters from the three species. Among them, 14 clusters, responsible for production of coelibactin, coelichelin, gray spore pigment, THN, bacteriocin, ectoine, indole, melanin, two types of NRPS-independent siderophres, and four types of terpenes are conserved among the three species, while additional five clusters for phenolic lipid, prodiginine, nonribosomal peptide, lantipeptide, and terpene syntheses are shared by two species. Coelibactin and coelichelin are iron-chelating molecules, known as siderophores, that are involved in uptake of ferric iron^17^. Like gray spore pigment and melanin, THN is involved in pigmentation, as it is used in melanin formation^18^. Pigment production is often examined in taxonomic studies^19^. Phenolic lipids are components of the cell wall, and are involved in resistance to β-lactam antibiotics by affecting the characteristics and rigidity of the cytoplasmic membrane/peptidoglycan^20^. Ectoine is an osmolyte and involved in protection against extreme osmotic stress^21^. Therefore, many of the conserved/shared gene clusters identified in this study are physiologically and/or taxonomically important. The remaining 33 smBGCs are species-specific, with each of the three species containing different eleven specific clusters.

Unexpectedly, most of the gene clusters in *S. rubrogriseus* NBRC 15455^T^ are present also in *S. coelicolor* (correctly classified as *Streptomyces violaceoruber*)^22^ A3(2). As the sequence similarities in these regions are very high (>93%), we considered it possible that strains NBRC 15455^T^ and A3(2) might actually be the same species. To clarify this, we conducted *in silico* DDH analysis of the two genome sequences. The resulting estimated DDH value is 70.3% (67.3–73.2%), which is just on the borderline between two strains belonging to the same or different species, and the probability that the value exceeds 70% was calculated to be 78.9% (data not shown). Orthologs of the other *t1pks* cluster(s) and *t2pks-b* found in *S. rubrogriseus* NBRC 15455^T^ (Table 6) were not identified in *S. coelicolor* A3(2), while orthologs of SCO5073-SCO5092 (actinorhodin), SCO6826-SCO6827, SCO7669-SCO7671 (aromatic polyketide), SCO7221 (germicidin), SCP1.228c-SCP1.246 (methylenomycin), SCO0381-SCO0401, and SCO7700-SCO7701 (2-methylisoborneol) present in *S. coelicolor* A3(2), could not be identified in *S. rubrogriseus* NBRC 15455^T^. These findings indicated that strains NBRC 15455^T^ and A3(2) are likely to be separate species. Very recently, phylogenetic relationships among *Streptomyces* species were examined using multi-locus sequence analysis. The study showed that *S. violaceoruber* was distinct from *S. rubrogriseus*^23^, supporting our current conclusion.

Here, we have shown an example that actinomycetes strains belonging to the same species share a conserved set of smBGCs, whereas different species each harbor species-specific smBGCs in addition to some common ones even if the species are taxonomically close. Relationships between species and smBGCs in actinomycetes were reported by Doroghazi *et al*.^24^, Ziemert *et al*.^16^, and Seipke *et al*.^25^. As the study by Doroghazi *et al*. is a large-scale analysis for taxonomically diverse 840 actinobacterial strains encompassing many genera, they did not compare smBGCs between taxonomically close *Streptomyces* species. Ziemert *et al*. reported the diversity and evolution of PKS and NRPS gene clusters within the genus *Salinispora*. In contrast to rare actinomycetes such as *Salinispora*, relationships between species and smBGCs are less well elucidated in the genus *Streptomyces*. Seipke *et al*. showed strain-level diversity of smBGCs in *S. albus*. However, the strains were actually not *S. albus*^23^ and may not belong to a single species but be divided into two independent genomospecies whose *in silico* DDH value is less 70% (our unpublished data). As the genus *Streptomyces* includes many species, accumulation of data for more *Streptomyces* species is needed to clarify whether smBGCs are diverse at strain-level or conserved at species-level. As reported here, genome sequence-based analysis will provide more insight into relationships between *Streptomyces* species and their secondary metabolites.

## Methods

### Strains

*Streptomyces diastaticus* subsp. *ardesiacus* NBRC 15402^T^, *Streptomyces coelicoflavus* NBRC 15399^T^, and *Streptomyces rubrogriseus* NBRC 15455^T^ were obtained from the NBRC (Biological Resource Center, National Institute of Technology and Evaluation, Chiba, Japan) culture collection. *Streptomyces* sp. TP-A0882 has been deposited into the NBRC culture collection and registered as NBRC 110030^12^.

### Analysis of 16S rRNA gene sequences

The 16S rRNA genes were amplified using two universal primers, 9F and 1541R, and sequenced according to an established method^26^. EzTaxon-e was used for basic local alignment search tool (BLAST) analysis of the sequences^27^.

### Genome sequencing

Genomic DNA was prepared from each of the strains as described previously^28^. The prepared DNA was subjected to paired-end sequencing using the MiSeq sequencing system (Illumina, San Diego, CA, USA) as per the manufacturer’s instructions. The sequence redundancies for the three draft genomes were 74-128-fold. The sequence reads were assembled using Newbler v2.8 (454 Life Sciences, Branford, CT, USA) and subsequently finished using GenoFinisher^29^.

### *In silico* DDH

DNA-DNA relatedness values were estimated from the genome sequences using Genome-to-Genome Distance Calculator (GGDC) 2.1, available from the Deutsche Sammlung von Mikroorganismen und Zellkulturen (DSMZ) website (http://ggdc.dsmz.de/distcalc2.php)^30^.

### Analysis of NRPS and PKS gene clusters

Coding regions in the draft genome sequences were predicted using Prodigal v2.6^31^. NRPS and PKS gene clusters were determined as previously reported^9,10^. A BLASTP search was performed using the NCBI Protein BLAST program (https://blast.ncbi.nlm.nih.gov/Blast.cgi?PAGE=Proteins), in which the non-redundant protein sequence (nr) database was chosen as the Search Set. AntiSMASH^32^ was used to predict substrates for adenylation, acyltransferase, and CoA ligase domains.

### Analysis of the other secondary metabolite biosynthetic gene clusters

BGCs except for PKS and NRPS gene clusters in the draft genome sequences were searched using antiSMASH^32^.

### Nucleotide accession numbers

The draft genome sequences in this study were deposited in GenBank/EMBL/DDBJ under the accession numbers shown in Table 1.
